# Fasting Glucose Levels Correlate with Disease Severity of Guillain-Barré Syndrome

**DOI:** 10.1371/journal.pone.0145075

**Published:** 2015-12-18

**Authors:** Ying Wang, Guihong Li, Siyu Yang, Xiaoyi Gu, Xinyu Li, Mingyang Liu, Xiujuan Wu, Yun Guan, Rayomand Press, Jie Zhu, Hong-Liang Zhang

**Affiliations:** 1 Department of Neurology, the First Hospital of Jilin University, Changchun, China; 2 Norman Bethune Health Science Center, Jilin University, Changchun, China; 3 Shanghai Medical College, Fudan University, Shanghai, China; 4 Division of Neurology, Department of Clinical Neuroscience, Karolinska Institute, Stockholm, Sweden; 5 Department of Neurobiology, Care Sciences and Society, Karolinska Institute, Stockholm, Sweden; Hannover Medical School, GERMANY

## Abstract

**Objective:**

A potential association between diabetes and Guillain-Barré syndrome (GBS) has been indicated by a few case studies. We retrospectively analyzed the clinical features of a large cohort of GBS patients to explore the relationship between the level of fasting plasma glucose (FPG) obtained in the acute phase at admission and the severity of GBS.

**Methods:**

Three hundred and four GBS patients were divided into two groups, one with normal FPG and the other with high FPG levels according to the international standards of FPG.

**Results:**

The GBS disability scale score was positively, the Medical Research Council (MRC) sum score was negatively correlated to the level of FPG, but not to blood HBA1c or CSF glucose concentrations. A relatively higher FPG level was observed in older and younger GBS patients, and more often in those with cranial nerve involvement, autonomic deficit, dyspnea and ventilator dependence than in patients without these clinical characteristics. Importantly, higher levels of FPG at admission were associated with poorer short-term prognosis measured by the MRC sum score and the GBS disability scale at discharge.

**Conclusions:**

Our data demonstrates that FPG in the acute phase of GBS correlates with the severity of GBS and may predict the short-term prognosis of GBS.

## Introduction

Findings from several preliminary studies have indicated a potential association between blood glucose levels and Guillain-Barré syndrome (GBS) [1–4]. Some patients with GBS developed hyperinsulinemia or hyperglycemia during the paralytic phase of disease despite not having been treated with corticosteroids [1]. Requirement for short-term insulin therapy, increased body mass index and elevated basal glucose indicated that insulin resistance was the predominant feature of impaired glucose tolerance in GBS [1]. The aberrant immune response proves critical in the pathogenesis of both GBS and diabetes. Gangliosides are expressed in both the peripheral nervous system (the node of Ranvier and axolemma) and the islets, and auto-antibodies to gangliosides appear in both GBS and type 1 diabetes as well [4]. Peripherin is a type 3 intermediate filament protein expressing mainly in neurons of the peripheral nervous system. A large proportion of peripherin-IgG seropositive patients had autonomic dysfunction or endocrinopathy [5]. Cytokines play an important role in the pathogenesis and development of both GBS and diabetes [6, 7]. Furthermore, the prevalence of neuropathy in the patients with diabetes was about 30%, and up to 50% of patients will eventually develop neuropathy during the course of disease [8]. Diabetes damages peripheral nerves through various pathways [9]. Although accumulated evidence implies a potential association between GBS and diabetes [1–4], this relationship has not been explored systematically in a large cohort of patients. Thus, we retrospectively analyzed the clinical manifestations of GBS patients and explored the relationship between the level of blood glucose and disease severity of GBS.

## Subjects and Methods

This study was approved by the ethics committee of the First Hospital of Jilin University, Changchun, China. Though written informed consent was not obtained, patient information was anonymized and de-identified.

### Study subjects

**Fig 1:**
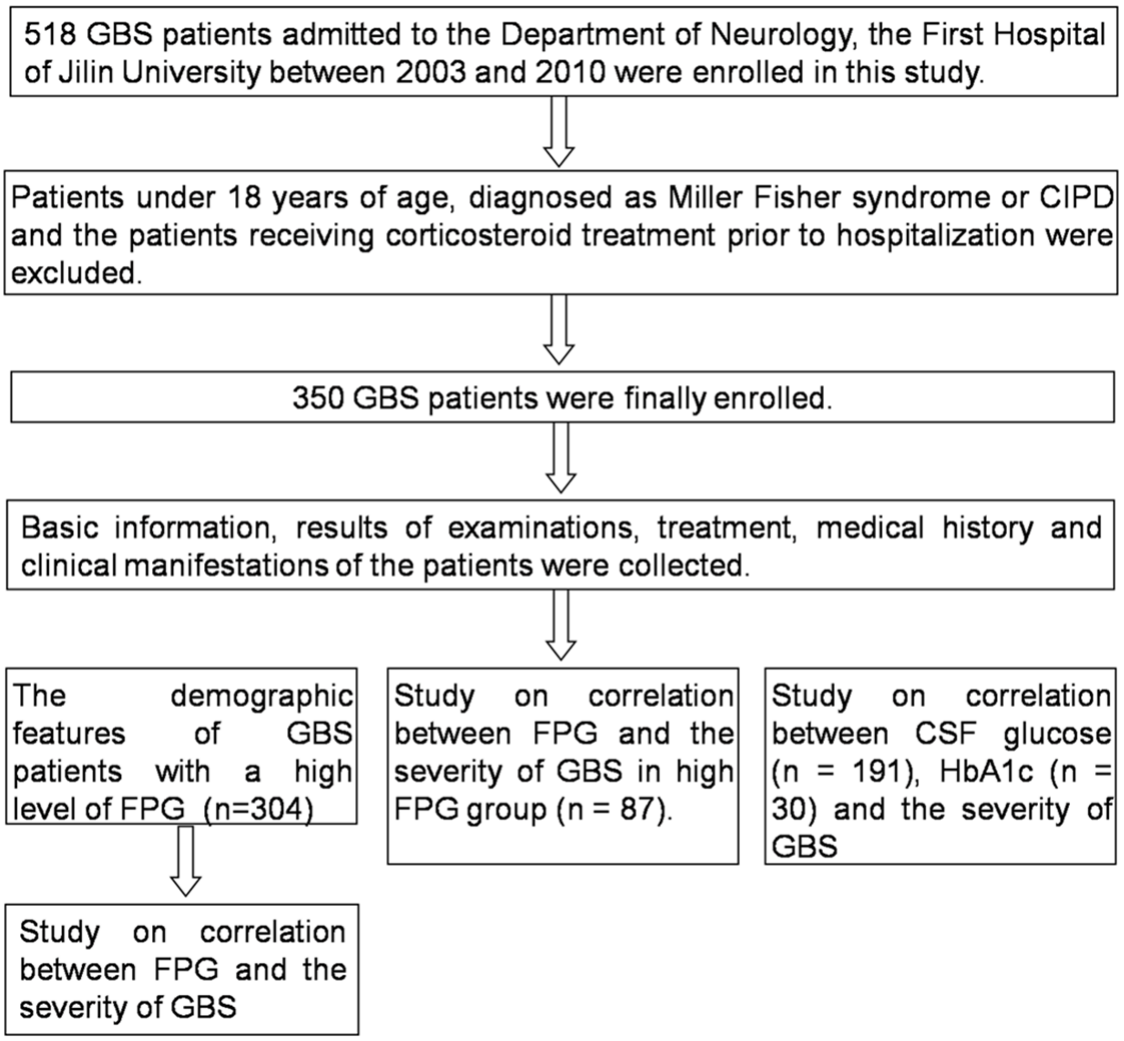
Flow chart of subject enrollment. This study was based on a database comprising 518 consecutive GBS patients. Patients under 18 years of age, diagnosed as Miller Fisher syndrome or CIPD and who received corticosteroid treatment prior to hospitalization were excluded. 350 GBS patients were finally enrolled.

This study was based on a cohort of 518 consecutive GBS patients admitted to the Department of Neurology, the First Hospital of Jilin University from 2003 till 2010. Patients under 18 years of age were ruled out because of their distinct clinical characteristics from adult patients [10]. Furthermore, patients diagnosed with Miller Fisher syndrome or chronic inflammatory demyelinating polyneuropathy (CIDP) and those who had received corticosteroid treatments prior to hospitalization were excluded. Finally 350 GBS patients were enrolled. Demographics, clinical symptoms, neurological examination, laboratory findings and treatment were collected (Fig 1).

### Evaluation of disease severity and functional impairments

Motor function deficits of patients were described by the GBS disability scale, a widely accepted scale of disability for GBS patients ranging from 0 to 6 (as follows: 0: healthy state; 1: minor symptoms and capable of running; 2: able to walk 5 m or more without assistance but unable to run; 3: able to walk 5 m across an open space with help; 4: bedridden or chair-bound; 5: requiring assisted ventilation for at least part of the day; 6: dead [11]). Weakness in extremities was expressed using the Medical Research Council (MRC) sum score of six bilateral muscles in arms and legs, ranging from 0 (tetraplegic) to 60 (normal strength) [12]. The nadir of GBS was defined as the lowest MRC sum score [11, 12]. Mild weakness was defined as MRC sum score ≥ 55 and tetraplegic as MRC sum score of 0.

### Laboratory testing of glucose

Blood samples were drawn from fasting patients at 5:30–6:00 of morning. The levels of FPG in 304 GBS patients were determined using One Touch blood glucometer (Johnson & Johnson Company, USA). The range of reference value was from 3.9 mmol/L to 6.1 mmol/L. Hemoglobin A1c (HbA1c) was measured by DCA Vantage-HbA1c analyzer (Siemens, Eschborn, Germany), reference value range of which was 4.8% to 6.0%. All the laboratory data were acquired at admission. Lumbar puncture was performed in 191 of 350 patients 2–3 weeks after the onset of the disease. The range of reference value of glucose in CSF was 0.18 mmol/L to 0.58 mmol/L.

### Statistical analysis

Statistical analysis was performed with SPSS version 18.0 software (SPSS, IBM, West Grove, PA, USA). The Chi-square or Fisher exact tests were used for testing differences in proportion, student-*t* test for normal continuous variable and Mann-Whitney U test for qualitative variable. Correlations were expressed by the Pearson rank correlation coefficient (r) or the Spearman rank correlation coefficient (rs). For all statistical tests, a two-side *p* value < 0.05 was regard as statistically significant.

## Results

### Clinical features of the patients in the high FPG group

**Fig 2:**
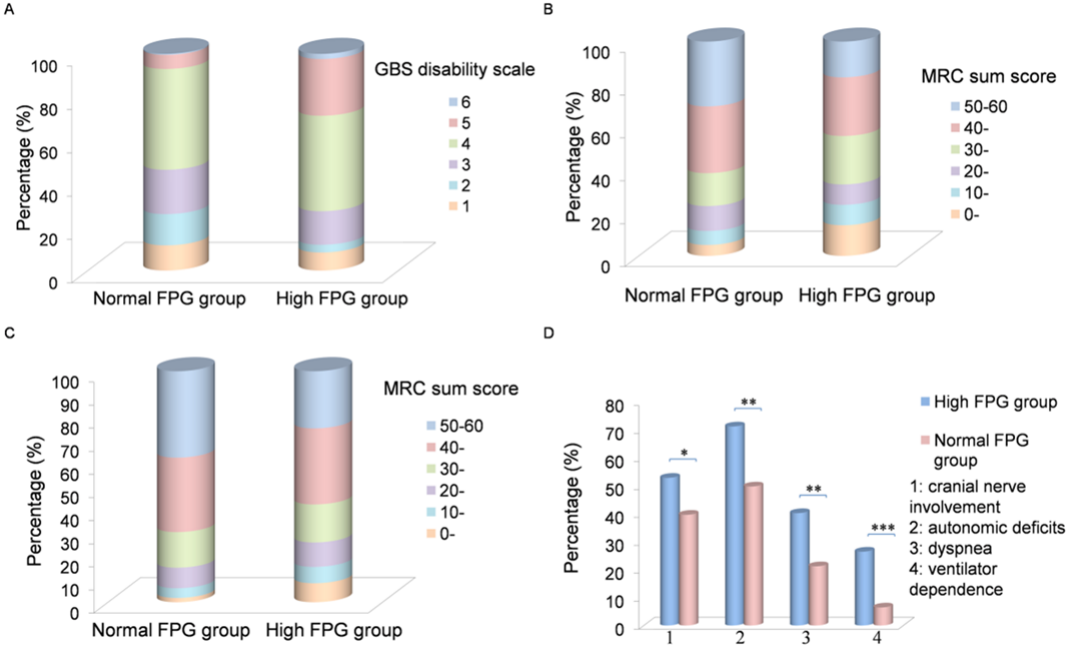
Clinical features of the patients in the high FPG group. (**A**) 304 subjects were divided into two groups. 217 patients whose FPG concentration was within the normal range (3.9–6.1 mmol/L) fell into the normal FPG group and the other 87 patients with a FPG concentration more than 6.1 mmol/L were put into the high FPG group. The GBS disability scale scores at nadir from 1 to 6 corresponded to 11.62%, 14.42%, 20.47%, 46.51%, 6.51% and 0.47% in the normal FPG group and to 8.33%, 3.57%, 15.48%, 44.05%, 26.19% and 2.38% of all patients in the high FPG group. The GBS disability scale score in the high FPG group was higher than that in the normal FPG group (*p* = 0.002). (**B**) Weakness in extremities was expressed using the MRC sum score of six bilateral muscles in arms and legs, ranging from 0 (teraparalytic) to 60 (normal strength). The MRC sum score for each level from 0–10 to 51–60 corresponded to 5.11%, 6.51%, 11.63%, 15.35%, 31.63% and 29.77% in the normal FPG group, while it was 14.30%, 9.52%, 9.52%, 22.61%, 27.38% and 16.67% in the high FPG group, indicating that the MRC sum score was rather lower in the high FPG group (*p* = 0.000). (**C**) The MRC sum score at study entry for each level from 0–10 to 51–60 corresponded to 1.87%, 4.21%, 8.88%, 15.42%, 32.24% and 37.38% in the normal FPG group, while it was 8.24%, 7.06%, 10.59%, 16.47%, 32.94% and 24.71% in the high FPG group. (**D**) Patients in the high FPG group had a tendentiousness of cranial nerve involvement, autonomic deficits, dyspnea, and ventilator dependence. The percentages of cranial nerve involvement, autonomic deficits, dyspnea and ventilator dependence were 25.12%, 39.63%, 49.77%, 21.20% and 6.45% in the normal FPG group, while it was 37.93%, 52.87%, 71.26%, 40.23% and 26.44% in the high FPG group (*p* < 0.05). *p < 0.05, **p < 0.01, *** *p* < 0.001.

Three hundred and four subjects enrolled in FPG study were divided into two groups (Fig 1). Two hundred and seventeen patients whose FPG concentration was within the reference value ranging from 3.9 mmol/L to 6.1 mmol/L were designated into the normal FPG group and the other 87 patients with an FPG concentration higher than 6.1 mmol/L into the high FPG group. The clinical features of the patients in the high FPG and normal FPG group are presented in Table 1. The severity of motor deficits significantly differed between the two groups (Fig 2A–2C). Additionally, patients in the high FPG group had a tendency of developing cranial nerve involvement, autonomic deficits (labile pulse rate or blood pressure), dyspnea and ventilator dependence (*p* < 0.05) (Fig 2D). In the high FPG group, symmetric weakness appeared in almost all patients: 75 (89.29%) had exactly the same MRC sum score at both sides and weakness at nadir ranged from mild severity in 10 (11.90%) to tetraplegic (the MRC sum score of 0) in 11 (13.10%) patients. MRC sum score at nadir was significantly higher than that in patients with decreased reflexes (*p* = 0.058). Moreover, CSF routine examination was performed in 39 patients and nerve conduction studies in 21 out of all 87 patients in the high FPG group (Table 1).

**Table 1:** Description of GBS patients in normal FPG glucose group and high FPG glucose group.

	Normal FPG group	High FPG group	*p* value
**Average of FPG concentration (mmol/L)**	5.11	7.53	
**Basic information**			
Male/female ratio	130/87	53/34	0.871
Age (years) ^a^	34 (24–44)	45 (34–55)	0.000
Duration in hospital (average)	15.06	23.40	0.002
**Symptoms of antecedent infection**	62.67% (136/217)	51.72 (45/87)	0.079
Diarrhea	25.35% (55/217)	19.54% (17/87)	0.282
Upper respiratory tract infection	26.27% (57/217)	21.84% (19/87)	0.420
Both	5.07% (11/217)	4.60% (4/87)	0.864
Others	6.00% (13/217)	5.74% (5/87)	0.935
**Movement system**			
GBS disability scale score at nadir			
1	11.62% (25/215)	8.33% (7/84)	0.471
2	14.42% (31/215)	3.57% (3/84)	0.008
3	20.47% (44/215)	15.48% (13/84)	0.324
4	46.51% (100/215)	44.05% (37/84)	0.701
5	6.51% (14/215)	26.19% (22/84)	0.000
6	0.47% (1/215)	2.38% (2/84)	0.192
MRC sum score at study entry ^a^	48 (36–55)	42 (24–49)	0.020
MRC sum score at nadir ^a^	42 (30–52)	36 (20–47.5)	0.003
Ataxia	6.45% (14/217)	4.60% (4/87)	0.536
**Cranial nerve involvement**	39.63% (86/217)	52.87% (46/87)	0.035
Oculomotor nerve	7.83% (17/217)	12.64% (11/87)	0.699
Abducent nerve	9.22% (20/217)	10.34% (9/87)	0.762
Facial nerve	23.50% (51/217)	35.63% (31/87)	0.031
glossopharyngeal-vagal nerves	27.19% (59/217)	42.53% (37/87)	0.009
**Sensory deficits**	39.17% (85/217)	35.63% (31/87)	0.556
**Reflexes**			
Decreased tendon reflexes	88.48% (192/217)	90.80% (79/87)	0.566
Pathological reflexes	8.76% (19/217)	3.45% (3/87)	0.106
Meningeal irritation sign	2.76% (6/217)	2.30% (2/87)	0.818
**Autonomic deficits**	49.77% (108/217)	71.26% (62/87)	0.001
**dyspnea**	21.20% (46/217)	40.23% (35/87)	0.001
**Ventilator dependence**	6.45% (14/217)	26.44% (23/87)	0.000
**Disturbances of consciousness**	2.30% (5/217)	10.34% (9/87)	0.005
**Lumbar puncture**			
Mean protein concentration (g/L)	0.96	1.27	0.162
Albumin-cytologic dissociations	72.90% (78/107)	79.49% (31/39)	0.418
Mean CSF IgG concentration (mg/L)	180.59	148.67	0.441
**Nerve conduction studies**			
Demyelinating group	47.62% (30/63)	66.67% (14/21)	0.130
Axonal group	34.92% (22/63)	28.57% (6/21)	0.593
Overlap group	17.46% (11/63)	4.76% (1/21)	0.279

^a^ Median (IQR)

Additionally, the high FPG group was divided into two subgroups: the high FPG group 1 and the high FPG group 2. The high FPG group 1 comprised 40 GBS patients with an FPG level more than 7.0 mmol/L which had a potential of impaired glucose tolerance while the high FPG group 2 comprised 47 patients with an FPG level ranging from 6.1 mmol/L to 7.0 mmol/L. Although no significant difference in clinical characteristics was detected between the two groups, patients in the high FPG group 1 tended to have a more severe clinical course measured by clinical manifestations and the MRC sum score/GBS disability scale (Table 2).

**Table 2:** Comparative study between high FPG group 1 and high FPG group 2.

	High FPG group 1	High FPG group 2	*p* value
**Basic information**			
Male/female ratio	23/17	30/17	0.719
Age (years) ^a^	46.5 (35.25–58)	44 (32–53.75)	0.466
**Weakness at nadir**			
GBS disability scale score			
1	5.26% (2/38)	10.87% (5/46)	
2	5.26% (2/38)	2.17% (1/46)	
3	15.79% (6/38)	15.22% (7/46)	
4	34.21% (13/38)	52.17% (24/46)	
5	34.21% (13/38)	19.57% (9/46)	
6	5.26% (2/38)	0% (0/46)	
MRC sum score at nadir ^a^	41 (26.75–48)	35 (18.75–46.50)	0.485
**Cranial nerve involvement**	60.00% (24/40)	46.81% (22/47)	0.219
**Sensory deficits**	30.00% (12/40)	40.43% (19/47)	0.312
**Decreased tendon reflexes**	92.50% (37/40)	89.36% (42/47)	0.721
**Autonomic deficits**	75.00% (30/40)	68.09% (32/47)	0.478
**Dispnea**	42.50% (17/40)	38.30% (18/47)	0.690
**Ventilator dependence**	35.00% (14/40)	19.15% (9/47)	0.095

^a^ Median (IQR)

### Association between the level of FPG and the severity of GBS

The MRC sum score, the GBS disability scale score and the duration in hospital correlated with the FPG level (rs = -0.115, *p* = 0.047; rs = 0.167, *p* = 0.004; rs = 0.186, *p* = 0.001). Linear regression/logistic regression model was used to depict the relation between FPG level and the MRC sum score/the GBS disability scale score at nadir. The FPG concentration was negatively/positively correlated to the MRC sum score/GBS disability scale (R^2^ = 0.023, p = 0.05; R^2^ = 0.14, p = 0.049). No correlation was found between the FPG level and the concentration of protein or IgG in CSF.

### Relationship between the levels of CSF glucose/HbA1c and the severity of GBS

**Fig 3:**
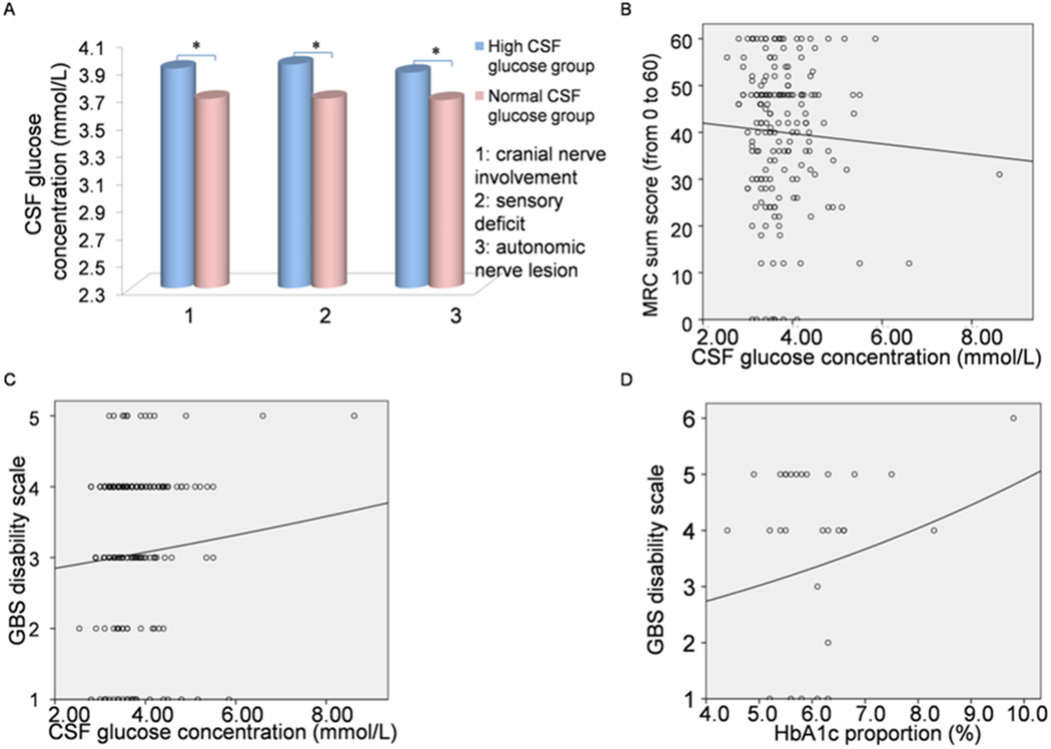
Relationship between the levels of CSF glucose/HbA1c and the severity of GBS. **(A)** 191 patients with available laboratory data of glucose in CSF were divided into two groups. Patients with CSF glucose concentration higher than 4.1 mmol/L fell into the high CSF glucose group, and the others into the normal CSF group. A high level of CSF glucose appeared in the patients who demonstrated cranial nerve involvement, hypoesthesia and autonomic deficits (3.90 mmol/L, 3.93 mmol/L and 3.87 mmol/L vs 3.68 mmol/L, 3.68 mmol/L and 3.67 mmol/L) (*p* < 0.05). **(B)** Relationships between CSF glucose concentration and the MRC sum score at nadir were measured by the linear regression (R^2^ = 0.03, *p* = 0.469). **(C)** The logistic regression was done to examine the relationship between the CSF glucose concentration and the GBS disability scale score (R^2^ = 0.003, *p* = 0.449). **(D)** The logistic regression was performed to explore the relationship between the HbA1c proportion and the GBS disability scale score (R^2^ = 0.029, *p* = 0.377).

One hundred and ninety one patients with available laboratory data of CSF glucose levels were divided into two groups. Patients with a CSF glucose concentration higher than 4.1 mmol/L fell into the high CSF glucose group, and the others into the normal CSF group. Analogously, 14 patients whose HbA1c proportion was over 6.0% were classified as the high HbA1c group, and the other 16 as the normal HbA1c group. Comparisons of clinical manifestations are shown in Fig 3A. The median MRC sum score in the high CSF glucose group at nadir was 42 and the IQR was 32–48. GBS disability scale from 1–6 was for 11.63%, 8.47%, 23.28%, 49.74%, 6.88% and 0%, respectively. Association between MRC sum score/GBS disability scale score and CSF glucose concentration was showed in Fig 3B and 3C. No statistical difference appeared in the study of HbA1c; however, patients with severe clinical manifestation tended to have a higher level of HbA1c (Fig 3D).

### Admission FPG predicts short-term prognosis

A relationship between admission FPG and discharge GBS disability scale/MRC sum score was explored as well. The median of duration between admission when FPG was measured and the peak of disease/discharge was 2 and 14 days with IQR of 0–4 and 9–21. At the time point of discharge, the median MRC sum score was 52 with IQR of 44–60 and it was roughly 16 scores improved compared to the MRC sum score at nadir. GBS disability scale corresponded to 31.92%, 22.70%, 16.31%, 25.98%, 2.13%, and 1.06% from 1 to 6 respectively and it was generally 2 point elevated compared to the peak. Interestingly, admission FPG was correlated to the GBS disability score and the sum MRC score in the lower extremities (rs = 0.147, *p* = 0.013; rs = -0.123, *p* = 0.041) at discharge. However, no correlation was found between FPG and the MRC sum score. Linear regression/logistic regression model showed a negative/positive association between admission FPG level and the sum of MRC score in the lower extremities/the GBS disability scale score at discharge (R^2^ = 0.010, *p* = 0.099; R^2^ = 0.016, *p* = 0.033).

## Discussion

GBS is an immune-mediated polyneuropathy. A few proposed studies have pointed to a potential association between diabetes and GBS [1–7]. Our retrospective analysis indicates that a high FPG level at admission is positively associated with the severity of GBS at discharge. Briefly, severe weakness, cranial nerve involvement, autonomic deficit and dyspnea are associated with a high level of FPG in the acute phase of GBS.

GBS and type 1 diabetes may share systemic immune pathways [6, 7]. Both GBS and type 1 diabetes can be triggered by vaccinations [13]. Gangliosides are present both in the nervous system and in the pancreas, while anti-ganglioside antibodies are found in serum of patients with GBS as well as those with type 1 diabetes [4]. Additionally, diabetes is associated with high levels of blood cytokines including IL-1β, IL-6, IL-12, IL-17, IL-18, TNF-α and IFN-γ, which mediate the physiological adaption of insulin production and insulin resistance [6]. These cytokines are involved in the pathogenesis of GBS as well [7, 14, 15]. Furthermore, an increased production of the C-reactive protein which is widely used as a biomarker for various infectious and inflammatory conditions, is observed in GBS patients [16]. As a consequence, the systemic inflammatory state in GBS may also lead to a temporal elevation of FPG. Taken together, we postulate that systemic inflammation may be the result shared by both GBS and diabetes, and that GBS might be aggravated by systemic inflammation. Both physiological and psychological stress may contribute to a metabolic disorder leading to hyperglycemia in GBS. Diabetes is a rare complication of GBS and diabetic ketoacidosis *per se* may precede GBS [1–3]. However, the CSF glucose levels were not associated with the severity of GBS in our study. The disparate breakdown of blood-CSF barrier may cause glucose leakage from the blood to the CSF to a different extent, which may bias our results.

Our study has limitations. Due to a retrospective nature of our study, we recorded FPG only once at admission and we did not monitor the fluctuation of FPG during the entire clinical course of GBS. Moreover, the measurement of HbA1c was done in relatively few patients and other important laboratory data for diabetes such as OGTT were unavailable. However, a large sample size and detailed clinical data of GBS patients support the validity of our conclusion.

In conclusion, our results point towards an association between FPG and severity of GBS. This association may be multi-factorial. Higher levels of pro-inflammatory cytokines, a heightened state of stress and related hypercortisolism and a higher level of autonomic dysfunction with an elevated sympathetic drive and related release of epinephrine and other stress hormones, comprise some of the possible explanations for this association. Further studies are warranted to identify the causal relationship between FPG and GBS.

## References

[1] Flax H, Matthews DR. Diabetes associated with Guillain-Barre syndrome. Diabetes Res 1990;14: 47–50. 2134666

[2] Kanemasa Y, Hamamoto Y, Iwasaki Y, Kawasaki Y, Honjo S, Ikeda H, et al A Case of Diabetic Ketoacidosis Associated with Guillain-Barré Syndrome. Intern Med 2011;50: 2201–2205. 21963741 10.2169/internalmedicine.50.5553

[3] Rouanet-Larriviere M, Vital C, Arne P, Favarel-Garrigues JC, Gin H, Vital A. Guillain-Barre syndrome occurring in two women after ketoacidosic comatose state disclosing an insulin-dependent diabetes mellitus. J Peripher Nerv Syst 2000;5: 27–31. 10780681 10.1046/j.1529-8027.2000.00122.x

[4] Misasi R, Dionisi S, Farilla L, Carabba B, Lenti L, Mario UD, et al Gangliosides and autoimmune diabetes Diabetes Metab Rev 1997;13: 163–179. 9307889 10.1002/(sici)1099-0895(199709)13:3<163::aid-dmr189>3.0.co;2-z

[5] Chamberlain JL, Pittock SJ, Oprescu AM, Dege C, Apiwattanakul M, Kryzer TJ, et al Peripherin-IgG Association with Neurologic and Endocrine Autoimmunity. J Autoimmun 2010;34: 469–477. 10.1016/j.jaut.2009.12.004 20061119 PMC2902873

[6] Donath MY, Dalmas É, Sauter NS, Böni-Schnetzler M. Inflammation in Obesity and Diabetes: Islet Dysfunction and Therapeutic Opportunity. Cell Metab 2013;17: 860–872. 10.1016/j.cmet.2013.05.001 23747245

[7] Lu MO, Zhu J. The role of cytokines in Guillain-Barré syndrome J Neurol. 2011;258: 533–548. 10.1007/s00415-010-5836-5 21104265

[8] Maser RE, Steenkiste AR, Dorman JS, Nielsen VK, Bass EB, Manjoo Q, et al Epidemiological correlates of diabetic neuropathy. Report from Pittsburgh Epidemiology of Diabetes Complications Study. Diabetes 1989;38: 1456–1461. 2620781 10.2337/diab.38.11.1456

[9] Zychowska M, Rojewska E, Przewlocka B, Mika J. Mechanisms and pharmacology of diabetes neuropathy—experimental and clinical studies. Pharmacol Rep 2013;65: 1601–1610. 24553008 10.1016/s1734-1140(13)71521-4

[10] Roodbol J, de Wit MC, Walgaard C, de Hoog M, Catsman Berrevoets CE, Jacobs BC. Recognizing Guillain-Barré syndrome in preschool children. Neurology 2011;76: 807–810. 10.1212/WNL.0b013e31820e7b62 21357832

[11] Hughes RA, Newsom-Davis JM, Perkin GD, Pierce JM. Controlled trial prednisolone in acute polyneuropathy. Lancet 1978;2: 750–753. 80682 10.1016/s0140-6736(78)92644-2

[12] Kleyweg RP, van der Meché FG, Schmitz PI. Interobserver agreement in the assessment of muscle strength and functional abilities in Guillain-Barré syndrome. Muscle Nerve 1991;14: 1103–1109. 1745285 10.1002/mus.880141111

[13] Tishler M, Shoenfeld Y. Vaccination may be Associated with Autoimmune Diseases. Isr Med Assoc J 2004;6: 430–432. 15274537

[14] Zhang HL, Zheng XY, Zhu J. Th1/Th2/Th17/Treg cytokines in Guillain-Barré syndrome and experimental autoimmune neuritis. Cytokine Growth Factor Rev 2013;24: 443–453. 10.1016/j.cytogfr.2013.05.005 23791985

[15] Zhang HL, Wu L, Wu X, Zhu J. Can IFN-γ be a therapeutic target in Guillain-Barré syndrome? Expert Opin Ther Targets 2014;18: 355–363. 10.1517/14728222.2014.882899 24479493

[16] Vaishnavi C, Kapoor P, Behura C, Singh SK, Prabhakar S. C-reactive protein in patients with Guillain-Barre syndrome. Indian J Pathol Microbiol.2014;57: 51–54. 10.4103/0377-4929.130897 24739831

